# Evidence mapping of Zhenwu decoction for chronic heart failure

**DOI:** 10.3389/fcvm.2026.1866697

**Published:** 2026-09-18

**Authors:** Boya Zhao, Yuerong Jiang, Yaxin Guo, Luying Chen, Yingzi Tan, Xirui Bao, Yuhang Fu, Yaojian Wang

**Affiliations:** 1 Xiyuan Hospital of China Academy of Chinese Medical Sciences, National Key Laboratory of TCM Syndrome, Beijing, China; 2 National Clinical Research Center of Chinese Medicine Cardiology, Beijing, China

**Keywords:** AMSTAR-2, chronic heart failure, evidence mapping, PRISMA, randomized controlled trial, Zhenwu decoction

## Abstract

**Objective:**

Chronic heart failure (CHF) remains associated with symptom burden, recurrent hospitalization, and reduced quality of life despite guideline-directed therapy. Zhenwu Decoction (ZWD), a classical traditional Chinese medicine formula, is used as adjunctive therapy for yang-deficiency and fluid-retention patterns. This evidence map aimed to characterize the volume, distribution, methodological quality, and evidence gaps in clinical research on ZWD for CHF.

**Methods:**

We searched CNKI, VIP, Wanfang, CMJ, SinoMed, PubMed, Web of Science, Embase, the Cochrane Library, ClinicalTrials.gov, ITMCTR, and ChiCTR from inception to 2 March 2025. Randomized controlled trials (RCTs), systematic reviews, and meta-analyses evaluating original or modified ZWD for CHF were eligible. Broad heart-failure terms were used for search sensitivity, but final eligibility was restricted to CHF. RCTs were assessed with the Cochrane risk-of-bias tool, and systematic reviews/meta-analyses were assessed with AMSTAR-2 and PRISMA 2020.

**Results:**

After exclusion of one acute heart failure study, the revised evidence base comprised 166 RCTs and 14 systematic reviews/meta-analyses. All included clinical studies were conducted in China and were generally single-center, small-sample, and short-duration trials with limited follow-up. Commonly reported outcomes included left ventricular ejection fraction, New York Heart Association functional class, and natriuretic peptide levels. The most frequently reported traditional Chinese medicine syndrome patterns were heart-kidney yang deficiency and yang deficiency with water retention. Important limitations included incomplete reporting of randomization, allocation concealment, blinding, protocol registration, follow-up, and adverse events. All included systematic reviews/meta-analyses were rated as critically low quality by AMSTAR-2.

**Conclusion:**

Current evidence suggests potential adjunctive signals for ZWD in CHF, but the certainty of evidence remains low. Future studies should use prospectively registered, adequately powered, multicenter RCTs with standardized syndrome differentiation, longer follow-up, clinically meaningful endpoints, and rigorous safety monitoring.

## 1 Introduction

Heart failure (HF) is a clinical syndrome caused by structural or functional cardiac abnormalities that impair ventricular filling or ejection. It is commonly characterized by dyspnea, fatigue, exercise intolerance, and fluid retention. With population aging and changes in cardiovascular risk profiles in China, HF has become an important cause of hospitalization, mortality, reduced quality of life, and socioeconomic burden (1–3).

Guideline-directed medical therapy can improve symptoms and outcomes, but many patients with chronic heart failure (CHF) continue to experience unstable symptoms, recurrent decompensation, and impaired daily function. In China, traditional Chinese medicine (TCM) is commonly used as an adjunct to conventional medical care. Conventional therapy is selected according to heart-failure phenotype and is intended to modify established cardiovascular and cardiorenal pathways with evidence-based effects on morbidity and mortality, whereas TCM treatment is generally individualized according to syndrome differentiation and used alongside, rather than instead of, standard therapy. For cardiovascular disease, previous evidence syntheses of Chinese herbal formulations used as adjuncts have reported signals for improvement in functional or surrogate outcomes and, in some analyses, fewer cardiovascular events or heart-failure rehospitalizations; however, methodological limitations, heterogeneity, and sparse safety and long-term endpoint reporting limit direct comparison with guideline-directed therapy (1, 4, 5, 7, 8). TCM formulas may influence symptom burden and functional status through multi-component actions related to fluid metabolism, neurohormonal activation, inflammation, oxidative stress, microvascular circulation, and myocardial energy metabolism; however, the certainty of clinical evidence varies and requires critical appraisal (6–9).

Zhenwu Decoction (ZWD) is derived from the Treatise on Cold Damage and Miscellaneous Diseases and comprises Aconiti Lateralis Radix Praeparata, Poria, Atractylodis Macrocephalae Rhizoma, Paeoniae Radix Alba, and Zingiberis Rhizoma Recens. In TCM theory, it is used to warm yang, promote water metabolism, strengthen the spleen, and reduce fluid retention. Here, heart-kidney yang deficiency denotes a TCM pattern in which reduced warming and transformative functions attributed to the heart and kidney are associated with manifestations such as fatigue, cold intolerance, reduced exercise capacity, and impaired fluid handling. Yang deficiency with water retention denotes a related pattern in which impaired fluid transformation is accompanied by oedema, weight gain, or other signs of fluid accumulation. These constructs are used for TCM pattern differentiation and treatment selection; they are not equivalent to Western heart-failure phenotypes or standalone biomedical diagnoses. Their clinical implication is that ZWD is traditionally selected for a clinically recognizable cluster of deficiency and fluid-retention manifestations, while variation in diagnostic criteria can introduce heterogeneity across trials (7, 8). ZWD has therefore attracted increasing clinical research interest for CHF. However, the distribution, quality, and limitations of this research field have not been systematically mapped.

Evidence mapping is a broad evidence-synthesis approach that describes the volume, characteristics, distribution, and gaps of research in a field. Unlike a conventional systematic review or meta-analysis, an evidence map does not primarily aim to generate a single pooled effect estimate. Instead, it visually and narratively summarizes where evidence is concentrated, where it is weak, and which questions require more rigorous study (10, 11). This approach is appropriate for ZWD in CHF because the literature is heterogeneous in study design, formula modification, treatment duration, outcomes, syndrome differentiation, and review quality.

## 2 Materials and methods

### 2.1 Literature search

The data were retrieved from China National Knowledge Infrastructure (CNKI), Wanfang Data Knowledge Service Platform, VIP Chinese Science and Technology Periodical Database, SinoMed, PubMed, Web of Science, Embase, the Cochrane Library, Chinese Medical Journals Full-text Database (CMJ), Chinese Clinical Trial Registry (ChiCTR), International Traditional Medicine Clinical Trial Registry Platform (ITMCTR), and ClinicalTrials.gov from inception to 2 March 2025. Chinese search terms included “真武汤”, “心力衰竭”, “慢性心力衰竭”, “心衰”, “心脏失代偿”, “心功能不全”, and “心衰竭”. English search terms included “Zhenwu decoction”, “Zhenwu Tang”, “heart failure”, “chronic heart failure”, “cardiac failure”, “cardiac insufficiency”, “heart decompensation”, “myocardial failure”, and “congestive heart failure”. Broad HF terms were used to maximize sensitivity because some records do not specify chronicity in the title or abstract, but final eligibility was restricted to CHF. The PubMed search combined MeSH and Title/Abstract terms, for example: (“Zhenwu decoction”[Title/Abstract] OR “Zhenwu Tang”[Title/Abstract] OR “Zhen Wu Tang”[Title/Abstract]) AND (“Heart Failure”[MeSH Terms] OR “heart failure”[Title/Abstract] OR “chronic heart failure”[Title/Abstract] OR “cardiac failure”[Title/Abstract] OR “cardiac insufficiency”[Title/Abstract] OR “congestive heart failure”[Title/Abstract]). Search expressions were adapted for each database. Dissertations, conference papers, trial registries, and other grey literature sources were eligible if they met the inclusion criteria. Literature search and screening were independently performed by two investigators.

### 2.2 Literature inclusion criteria

Eligible studies were publicly accessible RCTs, systematic reviews, or meta-analyses that evaluated ZWD as an intervention for CHF. The intervention had to use the original ZWD formula or a modified formula retaining the core components of Aconiti Lateralis Radix Praeparata, Poria, Atractylodis Macrocephalae Rhizoma, Paeoniae Radix Alba, and Zingiberis Rhizoma Recens. Eligible comparators included standard Western medical care, placebo, or standard care combined with placebo. Only Chinese- or English-language publications were included.

### 2.3 Literature exclusion criteria

Exclusion criteria were: (1) duplicate publications with identical data; (2) narrative reviews; (3) animal or *in vitro* studies; (4) studies with insufficient extractable data; (5) inaccessible full text; (6) studies in which ZWD was administered only to the control group; (7) trials in which the experimental group received additional TCM interventions, such as another herbal formula, Chinese patent medicine, acupuncture, or moxibustion, when the independent effect of ZWD could not be separated; (8) RCT protocols; (9) studies in which modified ZWD added or removed three or more herbal components from the core formula; and (10) studies focused on acute heart failure rather than CHF.

### 2.4 Literature screening and data extraction

Literature screening and data extraction were independently conducted by two investigators using a blinded approach. Disagreements were resolved through discussion or consultation with a third reviewer. References were managed with EndNote 21. After duplicate removal, titles and abstracts were screened according to predetermined criteria, followed by full-text eligibility assessment. Extracted items included author, year, title, study design, sample size, type of heart failure, TCM syndrome pattern, intervention and control regimens, ZWD composition or modification, treatment duration, follow-up, cardiac function classification, outcome measures, overall response rate, underlying disease, comorbidities, and adverse-event reporting.

### 2.5 Quality assessment

The quality of included RCTs was assessed with the Cochrane risk-of-bias tool, and systematic reviews/meta-analyses were assessed with AMSTAR-2. RCT risk-of-bias assessment was conducted at the study level because this evidence map aimed to describe the overall evidence landscape rather than produce outcome-specific pooled effects. For random sequence generation, low risk was assigned only when an adequate method, such as a random-number table or computer-generated sequence, was explicitly described. Merely stating “randomized” was not considered sufficient. Allocation concealment was rated as low risk only when central randomization, sealed opaque envelopes, or another adequate concealment method was reported. Blinding, incomplete outcome data, selective reporting, and other bias were judged according to the information available in each published report.

AMSTAR-2 was used to evaluate the methodological quality of systematic reviews and meta-analyses. The critical domains were items 2, 4, 7, 9, 11, 13, and 15. Overall confidence was rated as high, moderate, low, or critically low according to AMSTAR-2 guidance (12). Reporting quality was assessed using the PRISMA 2020 checklist, with each item scored as 0, 0.5, or 1 according to completeness of reporting.

### 2.6 Evidence mapping and classification rules

Evidence maps were used to summarize publication trends, intervention characteristics, outcome distribution, TCM syndrome patterns, methodological quality, reporting quality, and the reported direction of effects. “Beneficial” was assigned when a review reported statistically significant pooled effects favoring ZWD on at least two prespecified major outcomes, or on one major clinical outcome plus consistent supportive secondary outcomes, without a reported increase in serious adverse events. “Potentially beneficial” was assigned when the direction of effect favored ZWD for at least one major outcome but certainty was weakened by heterogeneity, imprecision, sparse safety reporting, inconsistent secondary outcomes, limited sample size, or critically low methodological quality. “No difference” was assigned when pooled results did not show a statistically significant or clinically meaningful difference. “Harmful” was assigned when ZWD was associated with worse efficacy outcomes or a higher rate of serious adverse events or toxicity. These categories describe reported direction of effect and do not indicate high certainty of evidence.

### 2.7 Protocol registration

This evidence map was not prospectively registered. The absence of protocol registration is acknowledged as a limitation because it may increase the risk of selective methodological decisions.

## 3 Results

### 3.1 Literature selection outcomes

The database and registry search identified 3,085 records. After removal of 1,834 duplicates, 1,251 records were screened by title and abstract, and 389 were excluded. Of 862 reports sought for retrieval, 7 were unavailable. A total of 855 full-text reports were assessed for eligibility. Reports were excluded because of inappropriate intervention (*n* = 603), non-eligible non-RCT design (*n* = 54), inappropriate topic (*n* = 17), or focus on acute heart failure (*n* = 1). The final evidence base comprised 180 studies, including 166 RCTs and 14 systematic reviews/meta-analyses. Figure 1 illustrates the PRISMA 2020 flow diagram.

**Figure 1:**
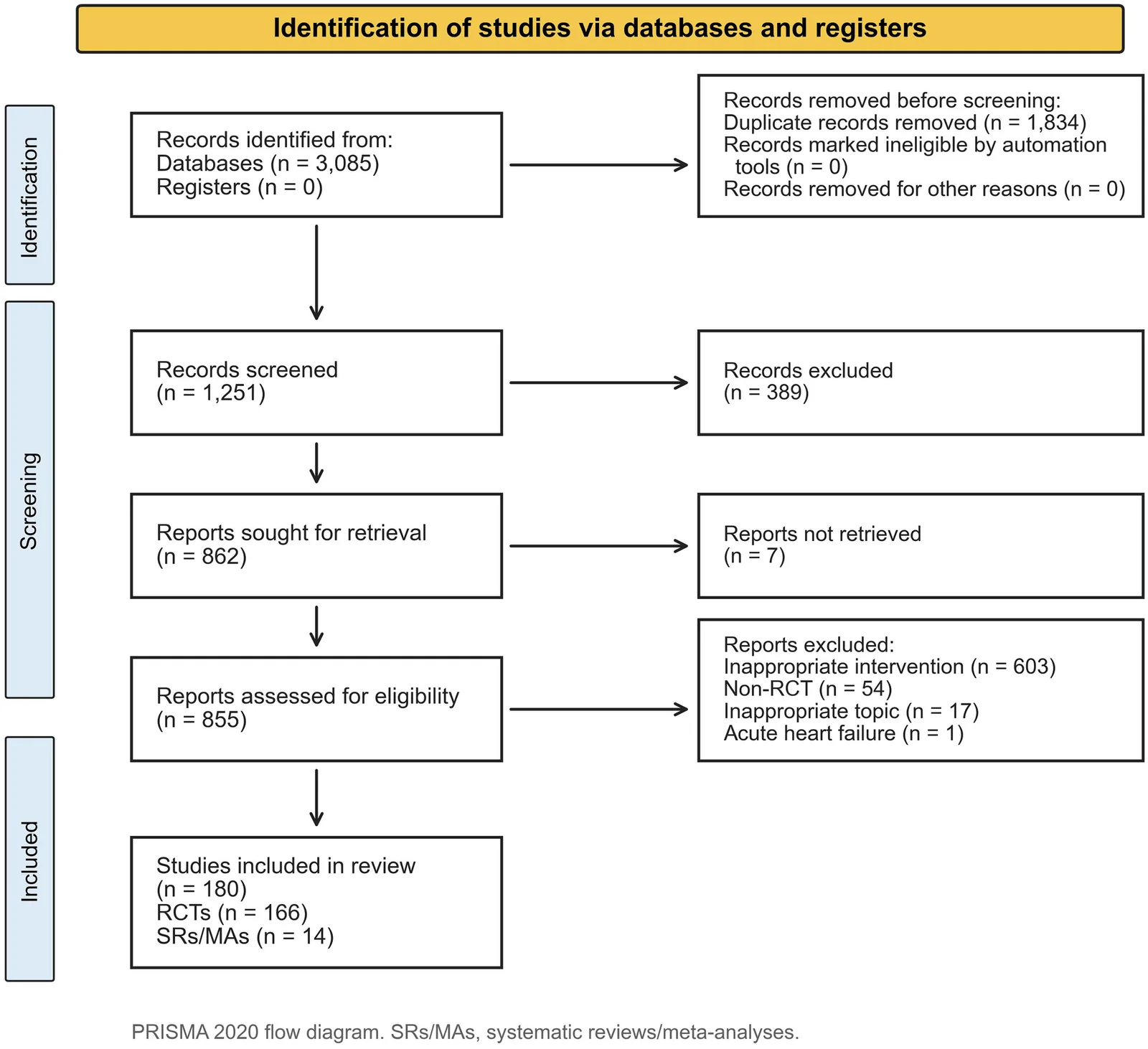
PRISMA 2020 flow diagram of study selection.

### 3.2 Baseline characteristics of included RCTs

The revised evidence base included 166 CHF RCTs, all conducted in China. The control groups received standard Western medical care or placebo, whereas the treatment groups received original or modified ZWD in addition to the control intervention. Most trials were small, single-center studies with short intervention durations and limited follow-up. Extracted study-level variables included sample size, CHF type, intervention regimen, treatment duration, main outcomes, follow-up, and adverse-event reporting.

### 3.3 Statistical analysis of heart failure types

After full-text reassessment, eligibility was restricted to CHF. The single study focused on acute heart failure after acute myocardial infarction was excluded from the revised evidence base. The remaining RCTs evaluated CHF populations or heart failure populations described as chronic, stable, or long-term disease.

Baseline LVEF values or LVEF subtype descriptions were reported in 103 studies. The original extraction counted some mixed or overlapping LVEF populations in more than one subtype, which caused subtype totals to exceed the number of studies reporting LVEF. In the revised presentation, LVEF categories should be reported as mutually exclusive groups: HFrEF, HFmrEF, HFpEF, mixed/overlapping LVEF, or unclear LVEF. The originally extracted subtype frequencies (HFrEF, *n* = 94; HFmrEF, *n* = 92; HFpEF, *n* = 20) are therefore reported as descriptive reporting frequencies rather than mutually exclusive study counts.

### 3.4 Annual publication trend

Between 1999 and 2025, the annual number of publications on ZWD for CHF fluctuated upward and peaked at 18 papers in 2020. The 2025 count includes records indexed only up to 2 March 2025 and should not be interpreted as a full-year estimate (Figure 2).

**Figure 2:**
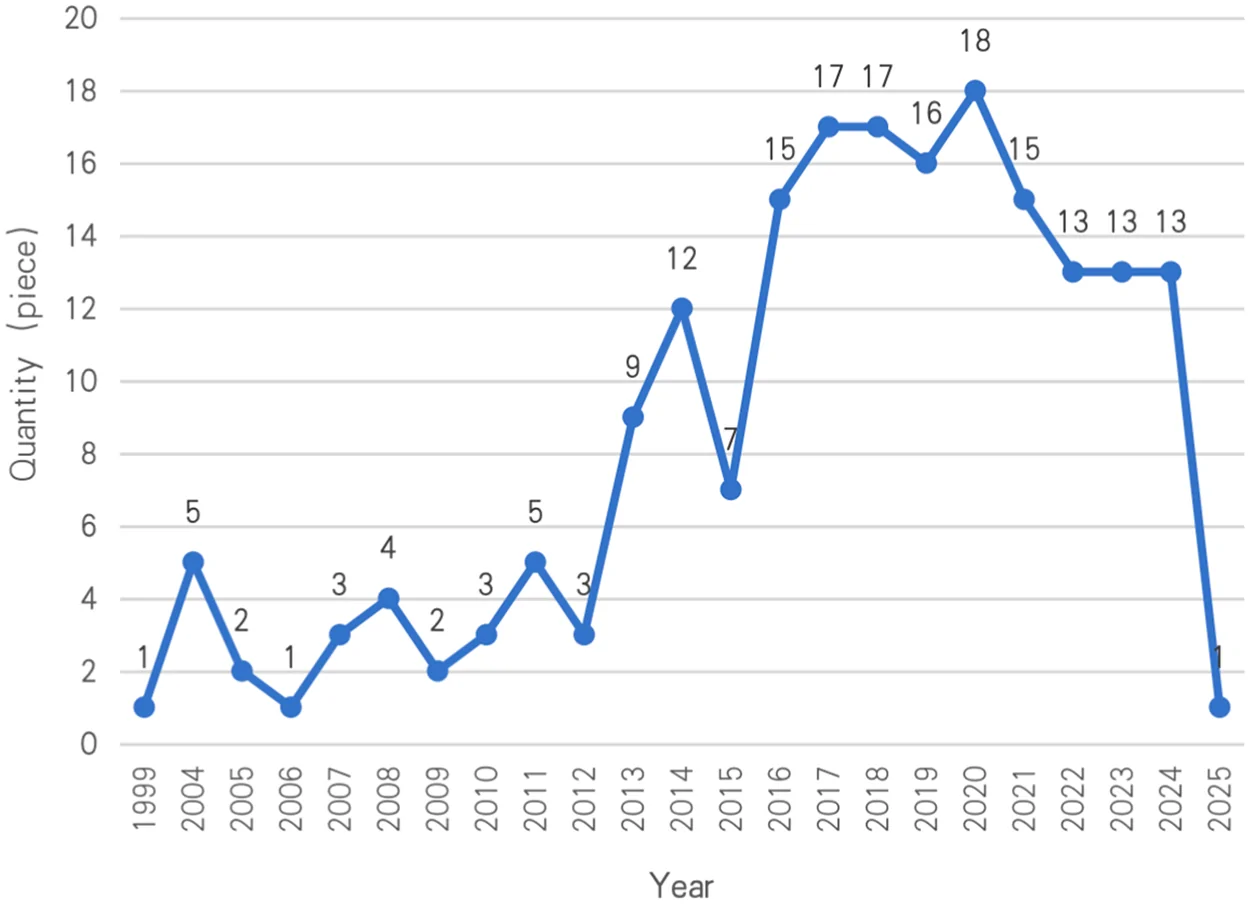
Annual publications on ZWD for CHF.

### 3.5 Journal publication volume

The included studies were published in 105 journals, 6 master's theses, 2 conference papers, 1 research website publication, and 1 newspaper article. Eighteen journals published at least three articles each. Twenty-eight articles were published in core journals, and three were indexed in SCIE journals. The top three journals by publication volume were Chinese Journal of Clinical Research on Traditional Chinese Medicine, Journal of Practical Traditional Chinese Medicine, and Chinese Journal of Integrative Medicine on Cardio/Cerebrovascular Disease.

### 3.6 Intervention duration and follow-up period

Thirteen studies did not report intervention duration. Among the remaining trials, most interventions lasted 15–30 days. Four studies lasted less than 7 days, 24 lasted 7–15 days, 79 lasted 15 days to 1 month, 30 lasted 5 weeks to 2 months, 12 lasted 3 months, and 5 exceeded 3 months. Most trials did not report post-treatment follow-up, limiting assessment of durability, rehospitalization, mortality, and delayed adverse events.

### 3.7 Pattern distribution analysis

Among the 166 CHF RCTs, 91 reported participants’ TCM syndrome patterns and described 11 unique patterns. Heart-kidney yang deficiency was the most frequent pattern (45/166, 27.1% of all CHF RCTs; 45/91, 49.5% of RCTs reporting syndrome patterns), followed by yang deficiency with water retention or water flooding (36/166, 21.7% of all CHF RCTs; 36/91, 39.6% of RCTs reporting syndrome patterns). The remaining patterns were reported infrequently and included spleen-kidney yang deficiency, qi deficiency with blood stasis and yang deficiency accompanied by water flooding, kidney yang deficiency, kidney yang decline, phlegm-fluid obstructing the lung, heart-kidney yang deficiency with yang deficiency and water flooding, yang deficiency with water flooding and spleen-stomach qi deficiency, yang deficiency with water flooding and blood stasis, and yang deficiency with water retention.

### 3.8 Heart function classification in included studies

In total, 118 articles documented the heart function classification; of these, 87 papers (73%) specified the number of patients in each category. Furthermore, among the 87 studies, 37 cases were categorized as NYHA Class I, 2,555 as Class II, 3,494 as Class III, and 1,570 as Class IV.

### 3.9 Distribution of primary diseases in included RCTs

The results also showed that 71 articles specified the primary heart failure diseases of enrolled patients, including coronary heart disease, hypertension, pulmonary heart disease, rheumatic heart disease, dilated cardiomyopathy, hyperthyroid heart disease, cardiomyopathy, valvular heart disease, arrhythmia, congenital heart disease, diabetes, and acute myocardial infarction. The top three of these diseases were coronary heart disease (*n* = 2,429), hypertension (*n* = 1,182), and pulmonary heart disease (*n* = 854).

### 3.10 Quality assessment of included literature

The included RCTs were assessed with the Cochrane risk-of-bias tool (Figure 3). The original reports frequently used the term “randomized”, but many did not provide enough detail to verify sequence generation or allocation concealment. Blinding of participants, personnel, and outcome assessment was rarely reported. The overall RCT evidence base therefore has important methodological and reporting limitations, including inadequate blinding, unclear allocation concealment, limited protocol availability, short follow-up, and incomplete safety reporting. The risk-of-bias results should be interpreted as reporting-based judgments rather than confirmation of high trial quality.

**Figure 3:**
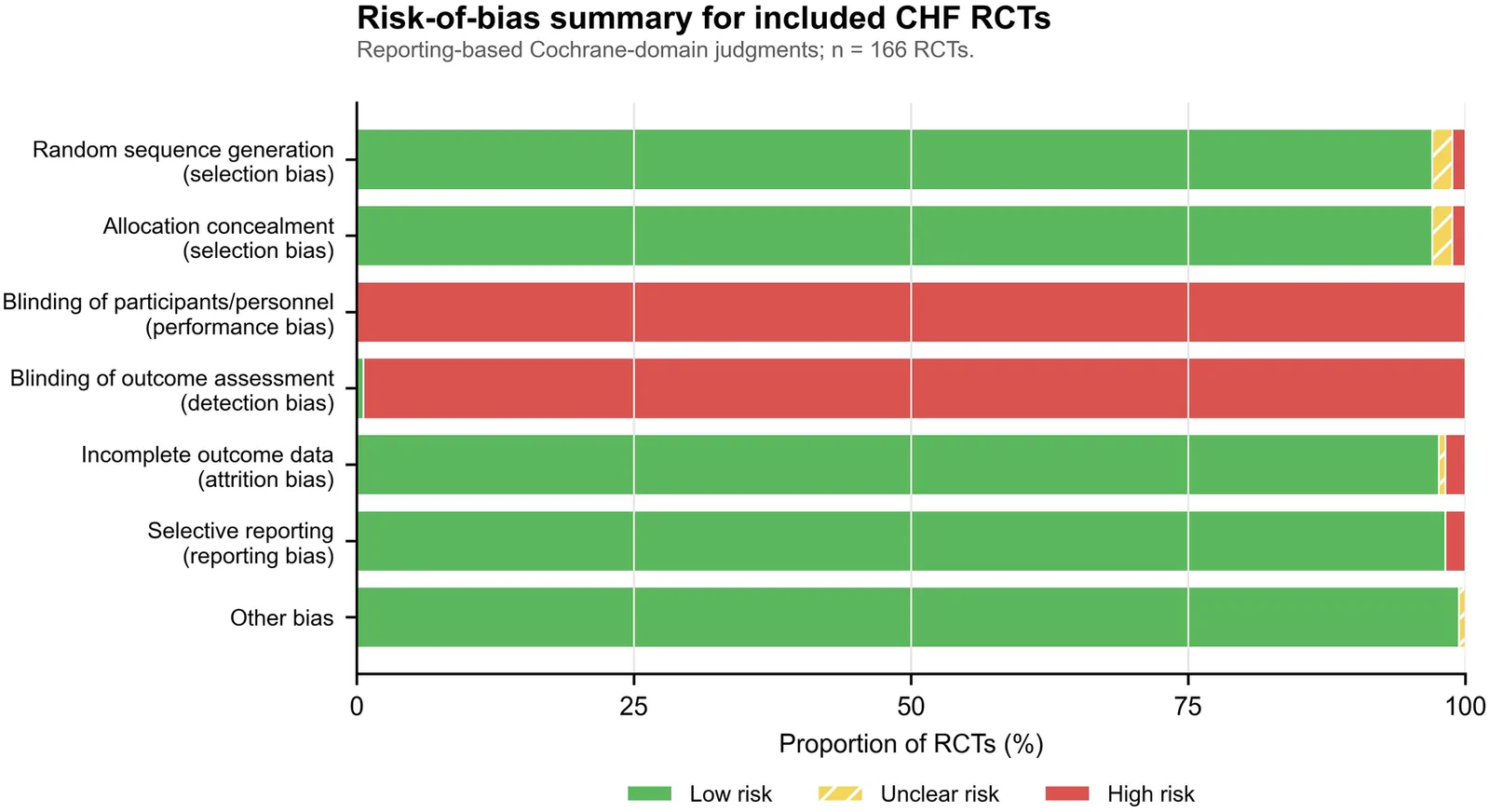
Risk-of-bias assessment of included RCTs.

### 3.11 Methodological quality assessment of systematic reviews

The AMSTAR-2 tool was used to evaluate the methodological quality of the 14 included systematic reviews/meta-analyses (13–26). All were rated as critically low quality. Common critical weaknesses included absence of a registered or prespecified protocol, incomplete search reporting, lack of a list of excluded studies with reasons, inadequate handling of risk of bias in primary studies, and insufficient assessment of publication bias (Figure 4).

**Figure 4:**
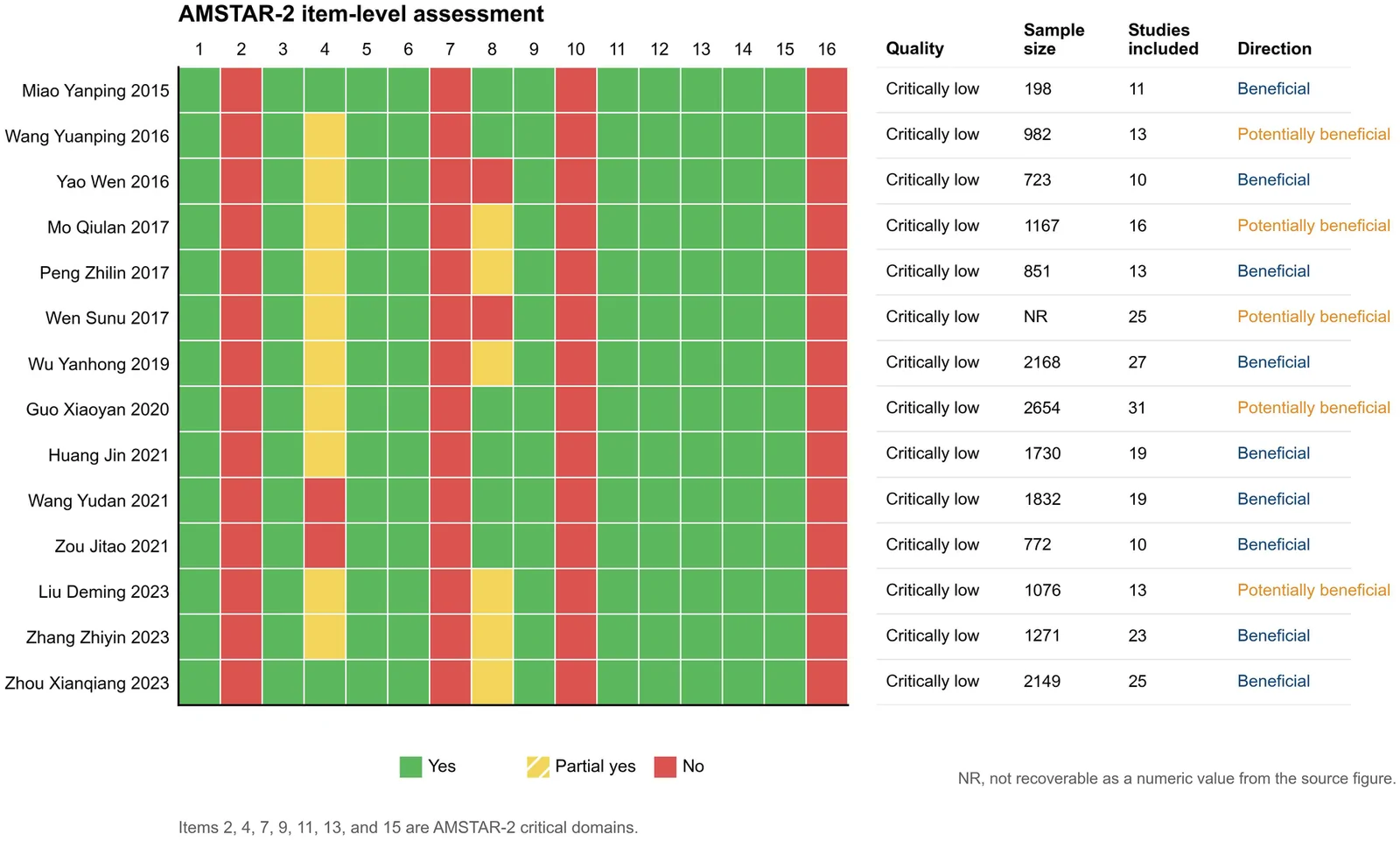
AMSTAR-2 item-level assessment of included systematic reviews/meta-analyses.

### 3.12 Evidence map

Among the 14 systematic reviews/meta-analyses, 9 were classified as reporting a beneficial direction of effect and 5 as reporting a potentially beneficial direction of effect according to the predefined rules. No review was classified as showing no difference or harmful effects. These labels describe the reported direction of effect only and should not be interpreted as high certainty. Because all included reviews were rated as critically low quality by AMSTAR-2, the direction of effect and methodological quality are presented separately in the evidence map (Figure 5).

**Figure 5:**
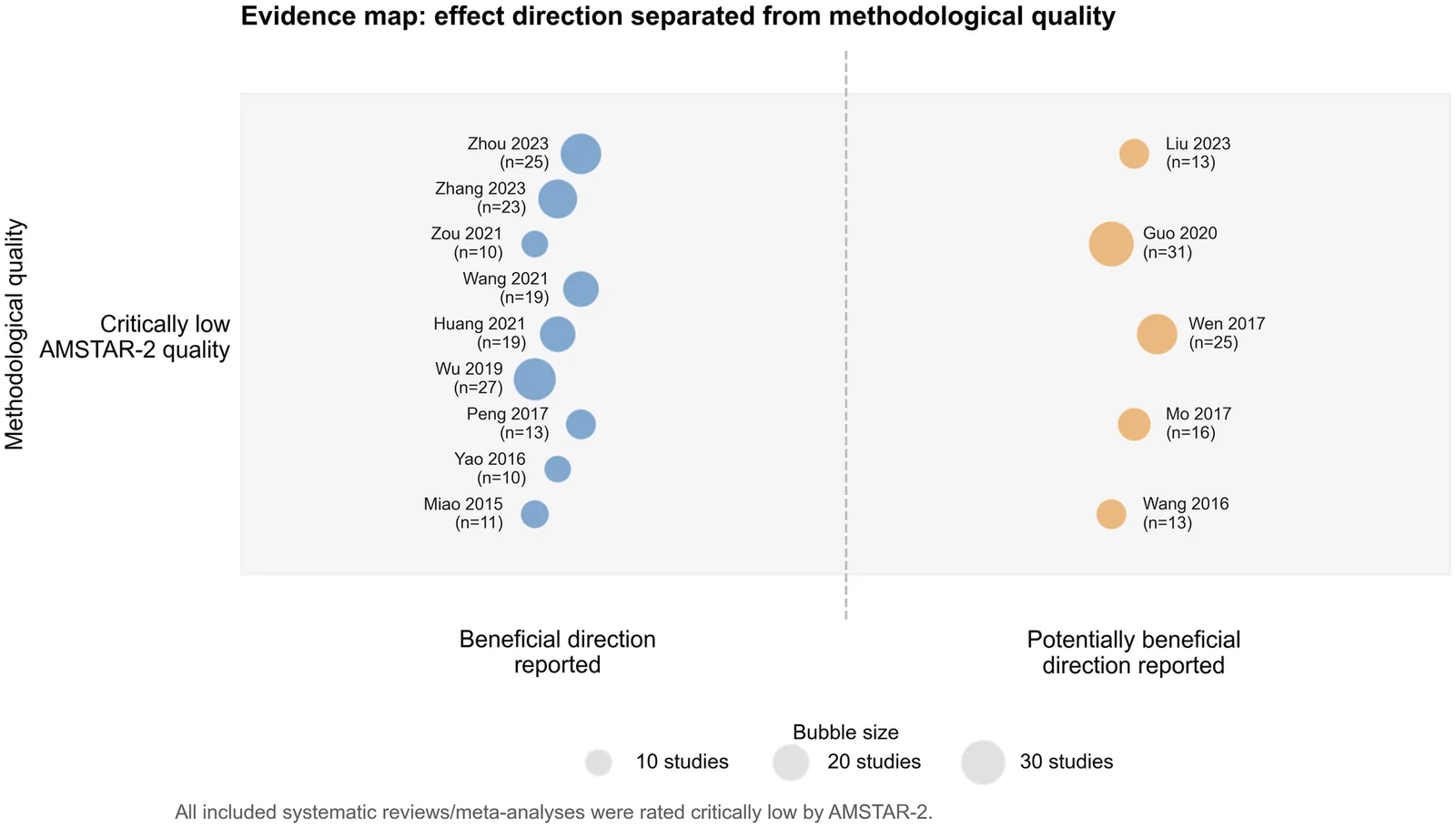
Evidence map of ZWD for CHF. Direction of effect and AMSTAR-2 methodological quality should be interpreted separately.

### 3.13 Quality assessment of systematic review reporting

Reporting quality of the included systematic reviews/meta-analyses was assessed with PRISMA 2020. Scores ranged from 13 to 20 out of 27. Commonly missing or incompletely reported items included protocol registration, detailed eligibility rationale, complete search strategies, certainty assessment, funding sources, conflicts of interest, and data availability. Total PRISMA scores should therefore be interpreted together with item-level reporting gaps.

## 4 Discussion

This evidence map summarizes the clinical literature on ZWD for CHF and shows that the field has expanded over time. The available literature suggests potential adjunctive signals for symptom-related and surrogate outcomes, but the certainty of evidence remains low. Nearly all clinical studies were conducted in China, most trials were single-center and small, intervention durations were usually short, and follow-up was rarely reported. These features limit generalizability and make it difficult to judge whether reported short-term improvements translate into durable benefits for hospitalization, mortality, or quality of life. This overall pattern is consistent with prior evidence syntheses of Chinese herbal adjuncts and ZWD-related interventions, which have reported possible benefits but also emphasized methodological limitations and uncertainty about long-term outcomes (1, 4, 5, 13–26).

The findings also highlight important methodological gaps. Many RCT reports did not adequately describe random sequence generation, allocation concealment, blinding, sample-size calculation, protocol registration, or adverse-event monitoring. These omissions increase the risk of bias and reduce confidence in the observed effects, consistent with concerns raised in prior TCM evidence syntheses and reporting-quality assessments (7, 8, 12–26). Future trials should prospectively register protocols, use adequate randomization and allocation concealment, apply placebo or matched controls when feasible, define CHF subtype and TCM syndrome pattern clearly, and report clinically meaningful endpoints with sufficient follow-up.

The distribution of TCM syndrome patterns was consistent with the traditional use of ZWD for yang deficiency with fluid retention, especially heart-kidney yang deficiency and yang deficiency with water retention. These patterns represent TCM constructs used to characterize deficiency-related symptoms and impaired fluid handling, rather than direct equivalents of Western heart-failure phenotypes (7, 8). However, syndrome differentiation was not uniformly reported, and formula modifications were heterogeneous. This variability limits the reproducibility of the intervention and complicates comparison across trials. A future evidence base would be strengthened by detailed reporting of herbal composition, permitted modifications, dose, decoction preparation, treatment duration, adherence, and co-interventions.

Safety evidence was also insufficient. ZWD contains Aconiti Lateralis Radix Praeparata, which requires careful processing, dosing, and monitoring. The absence of clearly reported toxicity signals in many clinical reports should not be interpreted as proof of safety because adverse-event definitions, monitoring procedures, serious adverse-event reporting, and follow-up were often incomplete; similar limitations have been noted in previous ZWD-related reviews and clinical reports (9, 13–26). Future studies should prespecify safety outcomes, actively monitor arrhythmia, neurological symptoms, gastrointestinal reactions, liver and kidney function, and other potential adverse events, and report serious adverse events transparently.

For future research, priority should be given to multicenter, adequately powered RCTs that use standardized diagnostic criteria, predefined CHF subtype categories, standardized syndrome differentiation, clearly specified ZWD formulations, core outcome sets, longer follow-up, and rigorous adverse-event monitoring. Outcome selection should move beyond short-term surrogate endpoints and include patient-centered and clinically meaningful endpoints such as quality of life, rehospitalization, and mortality, which are also emphasized in contemporary heart-failure guidance (1). Future systematic reviews should be prospectively registered, follow PRISMA 2020, provide complete search strategies and excluded-study lists, assess risk of bias transparently, and evaluate certainty of evidence (12–26).

## 5 Conclusion

In summary, this evidence map identified a growing body of clinical research on ZWD for CHF. Current studies suggest potential adjunctive benefits, particularly for symptom-related and surrogate outcomes in patients with heart-kidney yang deficiency or yang deficiency with water retention patterns. However, the certainty of evidence remains low because of small single-center trials, short treatment duration, insufficient follow-up, incomplete safety reporting, unclear risk-of-bias reporting, and critically low-quality systematic reviews/meta-analyses (13–26). ZWD should therefore be interpreted as a promising but insufficiently confirmed adjunctive intervention, consistent with its role alongside rather than in place of guideline-directed medical therapy (1, 7). Future research should prioritize prospectively registered, multicenter, adequately powered RCTs with standardized syndrome differentiation, transparent formula reporting, clinically meaningful endpoints, longer follow-up, and rigorous safety monitoring.

## Data Availability

The original contributions presented in the study are included in the article/Supplementary Material, further inquiries can be directed to the corresponding author.
