# Supplementary material for: Evidence mapping of Zhenwu decoction for chronic heart failure
Source: Front Cardiovasc Med. 2026 Sep 18;13:1866697. doi: 10.3389/fcvm.2026.1866697 (PMC13630523; doi:10.3389/fcvm.2026.1866697)
Supplement: Supplementary file 1 [file Datasheet1.docx]

**Supplementary Materials**

Evidence Mapping of Zhenwu Decoction for Chronic Heart Failure

# Supplementary Table S1

Complete search strategies are provided in the accompanying Excel workbook.

# Supplementary Table S2

Included systematic reviews/meta-analyses and reconstructed AMSTAR-2/evidence-map data.

# Supplementary Table S3

ZWD formula and modification eligibility criteria.

# Supplementary Table S4

Risk-of-bias domain summary for 166 CHF RCTs.

# Supplementary Table S5

AMSTAR-2 item-level judgments for 14 systematic reviews/meta-analyses.

# Supplementary Table S6

PRISMA 2020 reporting-quality gaps.

# Supplementary Table S7

Key outcome evidence summary.

# Supplementary Table S8

Figure data for annual publication trend.

The editable spreadsheet version is: Supplementary_materials_ZWD_CHF.xlsx
